# Action of an endo-β-1,3(4)-glucanase on cellobiosyl unit structure in barley β-1,3:1,4-glucan

**DOI:** 10.1080/09168451.2015.1046365

**Published:** 2015-06-01

**Authors:** Takao Kuge, Hiroki Nagoya, Theodora Tryfona, Tsunemi Kurokawa, Yoshihisa Yoshimi, Naoshi Dohmae, Kazufumi Tsubaki, Paul Dupree, Yoichi Tsumuraya, Toshihisa Kotake

**Affiliations:** ^a^ Life Science Materials Laboratory, Research and Development Division, ADEKA Corporation, Tokyo, Japan; ^b^ Division of Life Science, Graduate School of Science and Engineering, Saitama University, Saitama, Japan; ^c^ Department of Biochemistry, University of Cambridge, Cambridge, UK; ^d^ Global Research Cluster, RIKEN, Saitama, Japan; ^e^ Institute for Environmental Science and Technology, Saitama University, Saitama, Japan

**Keywords:** barley, cellobiosyl unit, endo-β-1,3(4)-glucanase: β-1,3:1,4-glucan, MALDI-ToF/ToF-/MS/MS

## Abstract

β-1,3:1,4-Glucan is a major cell wall component accumulating in endosperm and young tissues in grasses. The mixed linkage glucan is a linear polysaccharide mainly consisting of cellotriosyl and cellotetraosyl units linked through single β-1,3-glucosidic linkages, but it also contains minor structures such as cellobiosyl units. In this study, we examined the action of an endo-β-1,3(4)-glucanase from *Trichoderma* sp. on a minor structure in barley β-1,3:1,4-glucan. To find the minor structure on which the endo-β-1,3(4)-glucanase acts, we prepared oligosaccharides from barley β-1,3:1,4-glucan by endo-β-1,4-glucanase digestion followed by purification by gel permeation and paper chromatography. The endo-β-1,3(4)-glucanase appeared to hydrolyze an oligosaccharide with degree of polymerization 5, designated C5-b. Based on matrix-assisted laser desorption/ionization (MALDI) time-of-flight (ToF)/ToF-mass spectrometry (MS)/MS analysis, C5-b was identified as β-Glc-1,3-β-Glc-1,4-β-Glc-1,3-β-Glc-1,4-Glc including a cellobiosyl unit. The results indicate that a type of endo-β-1,3(4)-glucanase acts on the cellobiosyl units of barley β-1,3:1,4-glucan in an endo-manner.

## Graphical abstract

**Figure:**
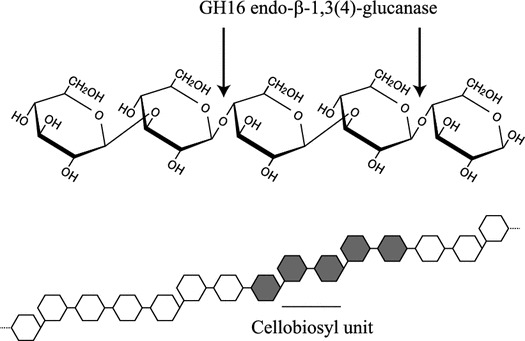

β-1,3:1,4-Glucan is a linear polysaccharide relatively abundant in young tissues and endosperm of grasses.^1^ ^)^ Although the mixed linkaged β-glucan does not exist in dicotyledonous plants, it has been also found in lichens such as *Cetralia islandica* and recently in horsetails (Equisetopsida).^2,3^ ^)^ β-1,3:1,4-Glucan is mainly consisted of cellotriosyl and cellotetraosyl units linked through single β-1,3-glucosidic linkages, though the proportion of cellotriosyl and cellotetraosyl units varies depending on the plant species. β-1,3:1,4-Glucan has also minor structures that are cellobiosyl units and long β-1,4-glucosyl stretches linked through a single β-1,3-glucosidic linkage, and continuous β-1,3-glucosyl residues.^4,5^ ^)^ The activity of β-1,3:1,4-glucan synthase has been seen in microsomal fractions prepared from young seedlings and endosperm in barley, maize, and rice.^6–8^ ^)^ To date, two glycosyltransferases, cellulose synthase-like F (CslF) and H (CslH), have been identified as components required for the synthesis of β-1,3:1,4-glucan in Poaceae.^9,10^ ^)^ However, it is still unknown whether β-1,3- and β-1,4-glucosyl residues are synthesized by single glycosyltransferase. In addition, the synthesis of cellotriosyl units was selectively inhibited by {3-[(3-cholamidopropyl)dimethylammonio]-1-propanesulfonic acid}, indicating the presence of at least two different glycosyltransferases synthesizing odd-numbered and even-numbered cellooligosaccharide units.^11,12^ ^)^ However, the precise mechanism for the synthesis of cellotriosyl and cellotetraosyl units and minor structures remains to be clarified.

β-1,3:1,4-Glucan undergoes degradation by endogenous hydrolases in young tissues and germinating seeds of Poaceae plants.^13,14^ ^)^ Endo-β-1,3:1,4-glucanase (EC 3.2.1.73) of Poaceae plants belonging to glycoside hydrolase (GH) family 17 is an enzyme specifically hydrolyzing the β-glucan in an endo-manner.^15^ ^)^ Higher plants also possess GH9 endo-β-1,4-glucanases (EC 3.2.1.4, cellulase) acting on the β-glucan in endo-manner.^16,17^ ^)^ In addition to the endo-acting enzymes, β-glucosidase (EC 3.2.1.21) and exo-β-glucanase (β-glucan exohydrolase, EC 3.2.1.58) hydrolyzing both β-1,3- and β-1,4-glucosidic linkages in exo-manner participate in the hydrolysis of β-1,3:1,4-glucan.^18,19^ ^)^ β-1,3:1,4-Glucan is also degraded by various enzymes secreted by fungi and bacteria in nature. Together with endo-β-1,3:1,4-glucanase and endo-β-1,4-glucanase,^4^ ^)^ endo-β-1,3(4)-glucanase (EC 3.2.1.6) takes part in the hydrolysis of β-1,3:1,4-glucan as an endo-acting enzyme. The enzyme has substrate specificity distinct from endo-β-1,3:1,4-glucanase and endo-β-1,4-glucanase, as it acts on both of β-1,3:1,4-glucan and β-1,3-glucan. Actually, an endo-β-1,3(4)-glucanase from *Phanerochaete chrysosporium*, PcLam16A, belonging to GH16 family acts on lichenin, β-1,3:1,4-glucan from *C. islandica*, liberating β-Glc-1,4-β-Glc-1,3-Glc (G4G3G) and also on β-1,3:1,6-glucan releasing G6G3G3G as the main products, respectively.^20^ ^)^ On the basis of three-dimensional (3-D) structure, the enzyme was revealed to accommodate both of β-1,3:1,6-glucan and β-1,3:1,4-glucan in its substrate-binding cleft by using different subsites +2, while it specifically recognizes the β-1,3-glucosidic linkage between the subsite −1 and −2.^21^ ^)^ It is probable that endo-β-1,3(4)-glucanases act not only on cellotriosyl and cellotetraosyl units but also cellobiosyl unit in β-1,3:1,4-glucan, but the action on cellobiosyl unit structure using a specific oligosaccharide remains to be examined.

In this study, we report that a GH16 endo-β-1,3(4)-glucanase from *Trichoderma* sp. can act on the cellobiosyl unit in barley β-1,3:1,4-glucan, while GH12 endo-β-1,4-glucanase from *Aspergilus niger* and GH17 endo-β-1,3-glucanase from barley cannot. The possible mechanism for the hydrolysis of the minor structure by the enzyme from *Trichoderma* sp. is discussed.

## Materials and methods

#### Materials

Carboxymethyl (CM)-cellulose, cellooligosaccharides, β-1,3:1,4-glucan from barley (high, medium, and low viscosity), GH16 endo-β-1,3(4)-glucanase from *Trichoderma* sp. (the commercial name is endo-β-1,3-glucanase), GH12 endo-β-1,4-glucanase (cellulase) from *A. niger*, and laminarioligosaccharides were purchased from Megazyme (Wicklow, Ireland). The purity of the enzymes was confirmed (Supplemental Fig. 1). Barley β-1,3:1,4-glucan, E70-S, was from ADEKA (Tokyo, Japan). Laminarin (β-1,3:1,6-glucan) from *Laminaria digitata* was from Sigma (St Louis, MO, USA). Recombinant barley endo-β-1,3-glucanases belonging to GH17, GI (rGI), and rGII,^22^ ^)^ were expressed in *Pichia pastoris* and purified by conventional chromatography (Supplemental Information, Supplemental Fig. 1).

#### Measurement of enzyme activity by reducing sugar assay

The activities of enzymes were measured using reaction mixtures (0.1 mL) consisting of the enzyme, 0.1% (w/v) polysaccharide, and 200 mM acetate buffer, pH 5.0. After incubation at 37 °C for the appropriate reaction time, the liberated sugars were determined reductometrically by the method of Nelson^23^ ^)^ and Somogyi.^24^ ^)^ One unit of enzyme activity liberates 1 μmol of reducing sugar per min. The concentration of protein was determined by the method of Bradford^25^ ^)^ using bovine serum albumin as the standard.

#### Analysis of endo-manner action on β-glucan

Digestion of β-1,3:1,4-glucan with enzyme was performed using a reaction mixture (total volume, 1 mL) consisting of the enzyme, 0.3% (w/v) β-1,3:1,4-glucan, and 50 mM 3-morpholinopropanesulfonic acid-NaOH buffer (pH 6.5). The apparent molecular weight (*M*_r_) of the β-glucan was estimated by high-performance liquid chromatography (HPLC) with a Shimadzu LC-10 A (Shimadzu, Kyoto, Japan) fitted with a refractive index detector (Shimadzu) and tandem columns (each 7.8 × 300 mm) of TSKgel G3000PW_XL_ and G2500PW_XL_ (Tosoh, Tokyo, Japan), equilibrated and eluted with 0.2 M potassium phosphate buffer (pH 6.9) at a flow rate of 0.8 mL/min and at 40 °C. The void volume (*V*_0_) and inner volume (*V*_i_) were determined with pullulan markers (Shodex Standard P-82; Showa Denko, Tokyo, Japan) and Glc.

#### Preparation of C5 oligosaccharides

One gram of barley β-1,3:1,4-glucan, E70-S, was digested with endo-β-1,4-glucanase from *A. niger* in 10 mM sodium acetate buffer (pH 4.5) at 37 °C for 24 h. The hydrolysate was lyophilized by freeze-dry and dissolved into 4 mL of water. Oligosaccharides released from the β-glucan were separated by gel permeation chromatography on a Bio-Gel P-2 column (26 mm × 925 mm, Bio-Rad). The *V*_0_ and *V*_i_ of the column were determined with dextran (Sigma) and Glc. Oligosaccharides were fractionated into C3, C4, and C5 fractions in order of increasing degree of polymerization (DP), which was determined by matrix-assisted laser desorption/ionization time-of-flight mass spectrometry (MALDI-ToF-MS) with a Bruker AutoflexIII (Bruker Daltonics, Bremen, Germany). C5 fraction was further fractionated into C5-a, -b, and -c by paper chromatography using Whatman 3MM filter paper with 6:4:3 (v/v/v) 1-butanol/pyridine/water (Supplemental Fig. 2). The sugar content of the fractions was determined by the phenol–sulfuric acid method using Glc as the standard.^26^ ^)^

#### Methylation analysis

For the analysis of sugar linkage, the oligosaccharide (approximately 100 μg) was subjected to the methylation analysis. Methylation was performed by the Hakomori method,^27^ ^)^ and the products were analyzed by gas liquid chromatography (GLC). GLC of neutral sugars as their alditol acetate derivatives was done with a Shimadzu gas chromatograph GC-6A equipped with a column (0.28 mm × 50 m) of Silar-10C, according to the method of Albersheim et al.^28^ ^)^

#### Action of enzymes on oligosaccharides

The action of the *Trichoderma* enzyme, rGI, and rGII on C4 and C5-b was analyzed using a reaction mixture (total volume, 20 μL) containing the enzyme, 0.1 mM oligosaccharide, and 50 mM sodium acetate buffer (pH 5.0). After incubation at 37 °C for 24 h, the sample was inactivated by heating. The reducing sugars liberated were coupled at their reducing terminals with *p*-aminobenzoic acid ethyl ester (ABEE) by the method of Matsuura and Imaoka.^29^ ^)^ The ABEE-derivatized sugars were analyzed on an HPLC system equipped with a TSKgel Amide-80 column (4.6 mm × 250 mm; Tosoh). The column was eluted with a linear gradient of CH_3_CN:water from 74:26 to 58:42 (v/v), for 40 min at a flow rate of 1 mL/min and 40 °C. ABEE sugars were monitored by a fluorescence detector model RF-10A_XL_ at 305 nm (excitation) and 360 nm (emission).

#### Structural analysis of oligosaccharide

For MALDI-ToF/ToF-MS/MS, per-methylation of glycans was performed using the NaOH slurry method described by Ciucanu and Kerek^30^ ^)^ using 1 mL of methyl iodide (Fluka, Buchs, Switzerland). Dry samples were resuspended in 100 μL of methanol and were kept at room temperature for MALDI-ToF/ToF-MS/MS analysis. Per-methylated methanol dissolved samples (5 μL) were mixed with 5 μL of 2,5-dihydroxybenzoic acid matrix {10 mg/mL dissolved in 50% (v/v) methanol} and 1 μL of the mixture was spotted on a MALDI target plate and analyzed by MALDI-ToF/ToF-MS/MS (4700 proteomics analyzer, Applied Biosystems, Foster City, CA, USA) as previously described.^31^ ^)^ High-energy MALDI collision-induced dissociation (CID) spectra were acquired with an average 10,000 laser shots/spectrum, using a high collision energy (1 kV). The oligosaccharide ions were allowed to collide in the CID cell with argon at a pressure of 2 × 10^−6^ Torr.

#### Polysaccharide analysis using carbohydrate gel electrophoresis

Products from the C5-b oligosaccharide by the *Trichoderma* enzyme were analyzed by polysaccharide analysis using carbohydrate gel electrophoresis (PACE). The derivatization of carbohydrates was performed according to previously developed protocols.^32^ ^)^ Carbohydrate electrophoresis and PACE gel scanning were performed as described by Goubet et al.^32^ ^)^

## Results

### Hydrolytic activity of endo-glucanases toward β-1,3:1,4-glucan

**Fig. 1.**
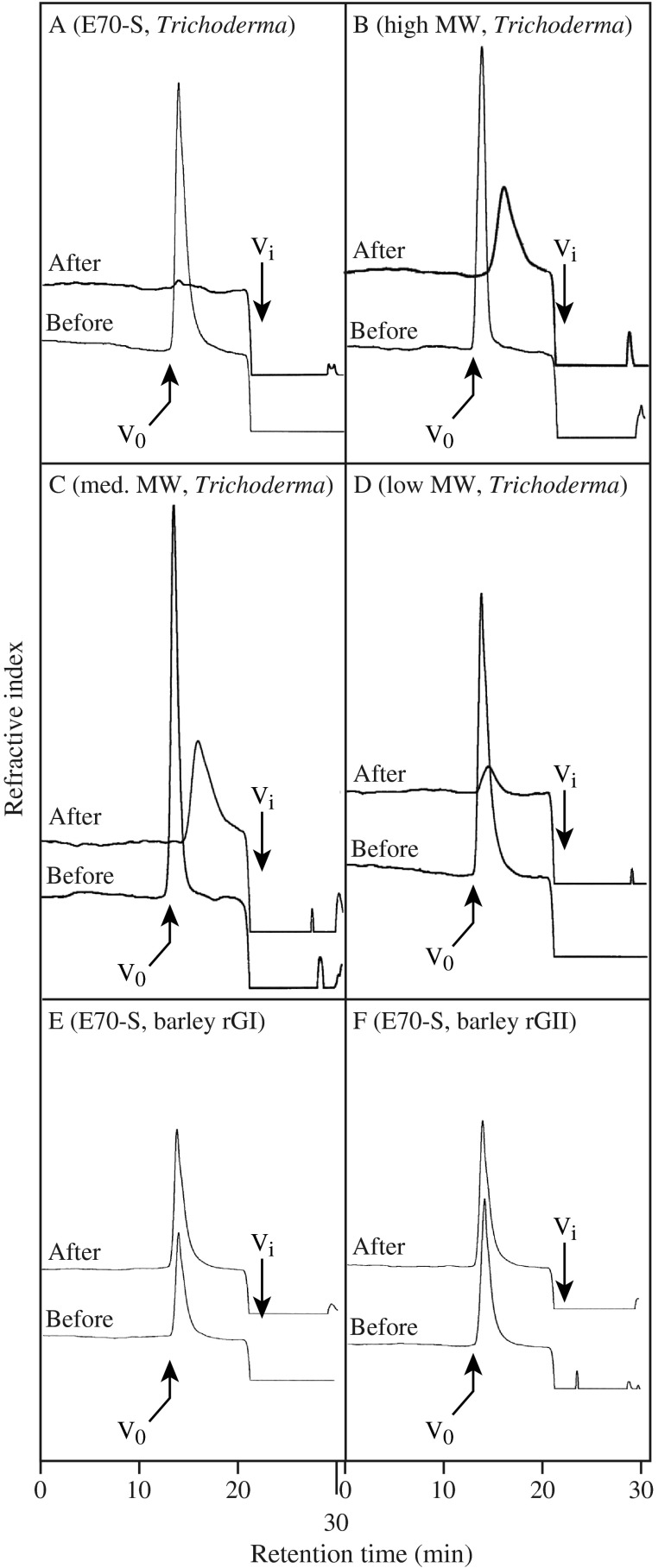
Breakdown of β-1,3:1,4-glucan by the *Trichoderma* endo-β-1,3(4)-glucanase.

Notes: A–D, β-1,3:1,4-Glucan, E70-S, and the β-glucans with high, medium, and low viscosity were digested with the *Trichoderma* endo-β-1,3(4)-glucanase, respectively. E and F, E70-S was treated with barley rGI and rGII, respectively. As the products were monitored by refractive index, smaller products eluted with salts at *V*_i_ could not be detected.

In this study, we analyzed the action of a commercial endo-β-1,3(4)-glucanase from *Trichoderma* sp. belonging to GH16 family and two recombinant barley endo-β-1,3-glucanases belonging to GH17 family, rGI and rGII,^22^ ^)^ on β-1,3:1,4-glucan. The *Trichoderma* enzyme purchased from Megazyme was identified as GH16 enzyme by MLADI-ToF/ToF-MS/MS analysis (data not shown). The rGI and rGII were expressed in *P. pastoris* and purified by conventional chromatography to homogeneity (Supplemental Information, Supplemental Fig. 1). First, the substrate specificity of the *Trichoderma* endo-β-1,3(4)-glucanase was confirmed by comparing with that of endo-β-1,3-glucanases rGI and rGII. The *Trichoderma* enzyme hydrolyzed laminarin, β-1,3:1,6-glucan from *L. digitata*, and barley β-1,3:1,4-glucan but hardly acted on CM-cellulose, proving that it has the characteristic of endo-β-1,3(4)-glucanase (Table 1). On the other hand, rGI and rGII failed to act on β-1,3:1,4-glucan. In addition, the endo-manner action of the *Trichoderma* enzyme on β-1,3:1,4-glucan was also confirmed. Consistent with the activity toward β-1,3:1,4-glucan determined by the reducing sugar assay, the *Trichoderma* enzyme decreased the apparent *M*_r_ of β-1,3:1,4-glucan, but rGI and rGII did not cause any change (Fig. 1).

**Table 1.** Substrate specificity of the enzymes.

Substrate^a^	rGI	rGII	*Trichoderma* enzyme
Laminarin (β-1,3:1,6-glucan)	100^b^	100	100
β-1,3:1,4-Glucan (high viscosity)	<0.1	<0.1	41
β-1,3:1,4-Glucan (medium viscosity)	<0.1	<0.1	9
β-1,3:1,4-Glucan (low viscosity)	<0.1	<0.1	9
CM-cellulose (β-1,4-glucan)	<0.1	<0.1	<0.1

^a^ The reducing sugars liberated from polysaccharide substrate by the enzyme were measured.

^b^ Activity is expressed in % of that toward laminarin.

### Isolation of oligosaccharides derived from the minor structure

**Fig. 2.**
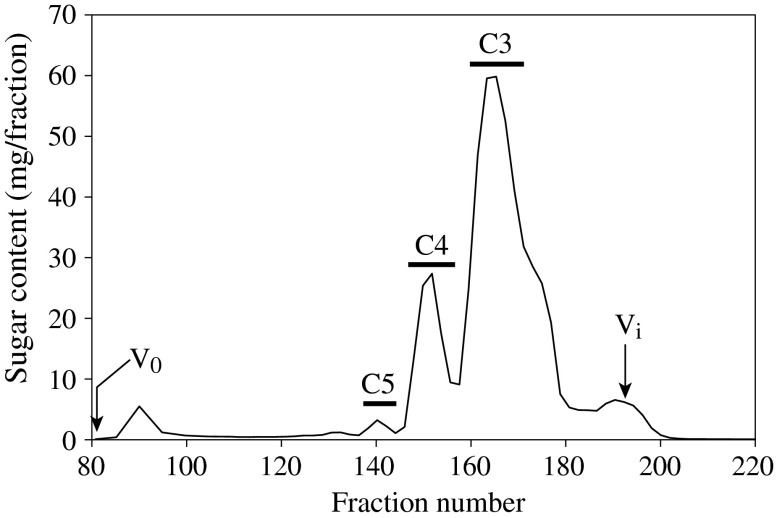
Separation of oligosaccharides released from β-1,3:1,4-glucan by endo-β-1,4-glucanase.

Notes: Barley β-1,3:1,4-glucan, E70-S, was hydrolyzed into oligosaccharides with endo-β-1,4-glucanase, and then subjected to gel permeation chromatography on a Bio-Gel P-2 column. *V*_0_ and *V*_t_ indicate void volume and total inner volume of the column, respectively. The horizontal bars indicate fractions pooled as C3, C4, and C5.

To identify the minor structure of β-1,3:1,4-glucan that is hydrolyzed by the *Trichoderma* endo-β-1,3(4)-glucanase, β-glucan E70-S was first hydrolyzed by GH12 endo-β-1,4-glucanase from *A. niger* in large scale, and the resulting oligosaccharides were fractionated into C3, C4, and C5 fractions by gel permeation chromatography (Fig. 2). The majority of oligosaccharides were fractionated into C3 and C4 fractions, which are presumably β-Glc-1,4-β-Glc-1,3-Glc (G4G3G) or β-Glc-1,3-β-Glc-1,4-Glc (G3G4G) derived from the cellotriosyl unit and β-Glc-1,4-β-Glc-1,4-β-Glc-1,3-Glc (G4G4G3G), β-Glc-1,4-β-Glc-1,3-β-Glc-1,4-Glc (G4G3G4G), or β-Glc-1,3-β-Glc-1,4-β-Glc-1,4-Glc (G3G4G4G) derived from the cellotetraosyl unit, respectively. The digestion also yielded a small amount (1.5% of total sugar) of C5 fraction presumably derived from the minor structures, on which the endo-β-1,4-glucanase does not act. MALDI-TOF-MS analysis revealed that C5 fraction has an oligosaccharide with DP 5 (Supplemental Fig. 3). The C5 fraction was further separated by paper chromatography into C5-a, -b, and -c (Supplemental Fig. 2). As C5-a and -c consisted of more than one oligosaccharides, C5-b was used for following analysis.

### Hydrolysis of C5-b oligosaccharides by Trichoderma endo-β-1,3(4)-glucanase

**Fig. 3.**
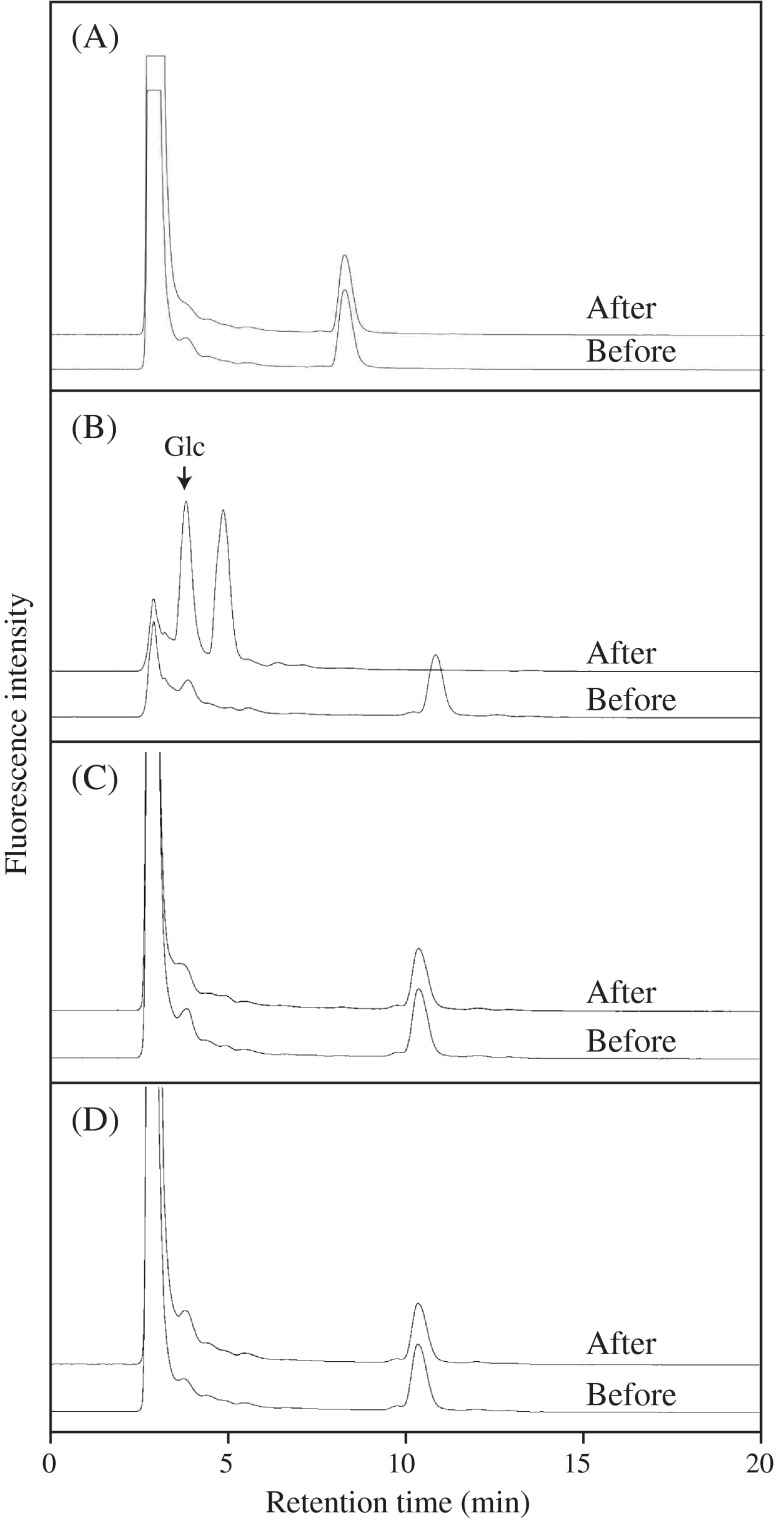
Action of the *Trichoderma* enzyme, barley rGI and rGII on oligosaccharides prepared from β-1,3:1,4-glucan.

Notes: A, the action of the *Trichoderma* enzyme on C4; B, the action of the *Trichoderma* enzyme on C5-b; C, the action of rGI on C5-b; and D, the action of rGII on C5-b were analyzed. Reducing sugars before and after enzyme reaction were derivatized with ABEE and analyzed on HPLC, respectively.

C5-b oligosaccharide was incubated with the *Trichoderma* enzyme, rGI, or rGII, and the hydrolysis was monitored on HPLC. While rGI and rGII failed to act on the substrate, the *Trichoderma* enzyme properly degraded C5-b into smaller oligosaccharides and glucose (Glc) (Fig. 3). The results indicate that C5-b structure is hydrolyzed by *Trichoderma* endo-β-1,3(4)-glucanase belonging to GH16, but not by *A. niger* endo-β-1,4-glucanase belonging to GH12 and barley endo-β-1,3-glucanases belonging to GH17, rGI, and rGII. On the other hand, C4 oligosaccharide derived from cellotetraosyl unit was not hydrolyzed by the *Trichoderma* enzyme at all.

### Linkage analysis of oligosaccharides

To determine the structure, C5-b oligosaccharide was subjected to methylation analysis for glucosidic linkages together with C3 and C4 oligosaccharides. In the analysis, sugars at reducing end were converted to their respective alditols before methylation of free OH groups. C4 oligosaccharide appeared to have nearly equal molecular ratio of terminal Glc (t-Glc), 3-linked Glc (3-Glc), 4-linked Glc (4-Glc), and 4-linked reducing-end Glc (4-Glcol), which coincides with the ratio obtained from G3G4G4G (Table 2). Similarly, C3 was identified as G3G4G. Compared with C3 and C4, C5-b had roughly two units of 3-Glc, indicating that C5-b is derived from a cellobiosyl unit or continuous β-1,3-glucosyl residues in β-1,3:1,4-glucan.

**Table 2.** Glucosidic linkage analysis of oligosaccharides derived from β-glucan, E70-S.

	t-Glc^a^	3-Glc	4-Glc	4Glcol^b^
C3	1.00	0.96	–^c^	1.46
C4	1.00	0.93	0.90	1.19
C5-b	1.00	1.72	1.14	0.53

^a^ Molar ratio is expressed based on either nonreducing (t-Glc) taken as 1.0.

^b^ Samples were methylated after reduction of their reducing ends with NaBH_4_.

^c^ Not detected.

### Structure of C5-b oligosaccharide

**Fig. 4.**
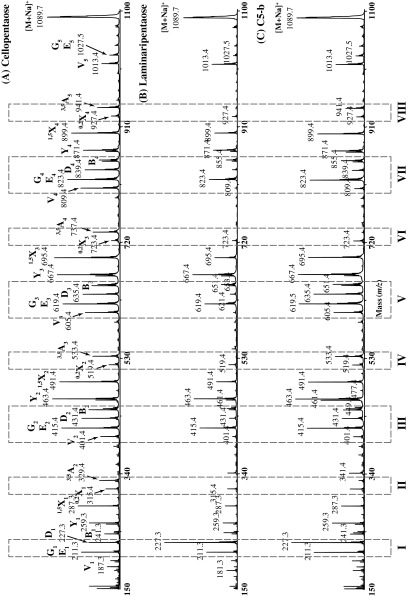
Structural characterization of C5-b oligosaccharide by MALDI-CID. Cellopentaose (A), laminaripentaise (B), and C5-b oligosaccharide (C) were per-methylated and analyzed by MALDI-CID.

Notes: The signals used for characterization were boxed numbered from I to VIII. Glucosidic and cross-ring fragments are identified according to the nomenclature of Domon and Costello.^33^^)^

For further structural analysis, C5-b oligosaccharide was also per-methylated and analyzed by high-energy MALDI-CID (Fig. 4, Supplemental Fig. 4).^33^ ^)^ However, because the direct annotation of the fragmentation spectrum was very ambiguous, we followed a comparative approach where the CID spectra of per-methylated cellopentaose, laminaripentaose, and C5-b oligosaccharide were simultaneously analyzed. In particular, we compared the relative proportions of various molecular ions in the corresponding spectra in order to decipher the structure of C5-b oligosaccharide. Comparing the CID spectra of cellopentaose and laminaripentaose, it becomes apparent that the intensity of the D_1_ “elimination ion” (*m/z* 227.3)^34^ ^)^ is higher than the neighboring E_1_ or G_1_ “elimination ions” (*m/z* 211.3)^35,36^ ^)^ when the nonreducing-end Glc is 1,3-linked to the second Glc residue (Fig. 4, panel I). In fact, the relative intensity of the *D*_1_ ion in the C5-b CID spectrum is higher than the *G*_1_ or *E*_1_ ion intensity, suggesting that the nonreducing-end Glc is linked via a 1,3-linkage to the second Glc residue. This is further supported by the absence of the ^3,5^A_2_ cross-ring fragment ion (*m/z* 329.4)^33^ ^)^ in the C5-b CID spectrum (Fig. 4, panel II) and the absence of the V_4_ “elimination ion” (*m/z* 809.4) (Fig. 4, panel VII). A strong ^3,5^A_3_ cross-ring fragment ion (*m/z* 533.4) over a ^0,2^X_2_ cross-ring fragment ion (*m/z* 519.4)^33^ ^)^ indicates that the second Glc is 1,4-linked to the middle Glc residue (Fig. 4, panel IV). This is further supported by the presence of a strong V_3_ “elimination ion” (*m/z* 605.4) (Fig. 4, panel V). From the comparison of the CID cellopentaose spectrum with the corresponding laminaripentaose spectrum, it becomes apparent that a weak V_2_ “elimination ion” (*m/z* 401.4) is indicative of a 1,3-linkage between the middle and penultimate from the reducing-end Glc residues (Fig. 4, panel III). Since the C5-b pre-methylated spectrum has a weak V_2_ ion, we conclude that in this oligosaccharide the middle Glc residue is 1,3-linked to the penultimate Glc residue. This is further supported by the absence of the ^3,5^A_4_ cross-ring fragment ion (*m/z* 737.4) (Fig. 4, panel VI) and the absence of the D_4_ “elimination ion” (*m/z* 839.4) (Fig. 4, panel VII). In Fig. 4, panel VIII, it is shown that a strong ^3,5^A_5_ cross-ring fragment ion (*m/z* 941.4) is indicative of a 1,4-linkage between the penultimate and the reducing-end Glc residues. Taken together, these data allow the C5-b oligosaccharide to be identified as β-Glc*p*-1,3-β-Glc*p*-1,4-β-Glc*p*-1,3-β-Glc*p*-1,4-Glc*p* (G3G4G3G4G).

### Products released from C5-b by Trichoderma enzyme

**Fig. 5.**
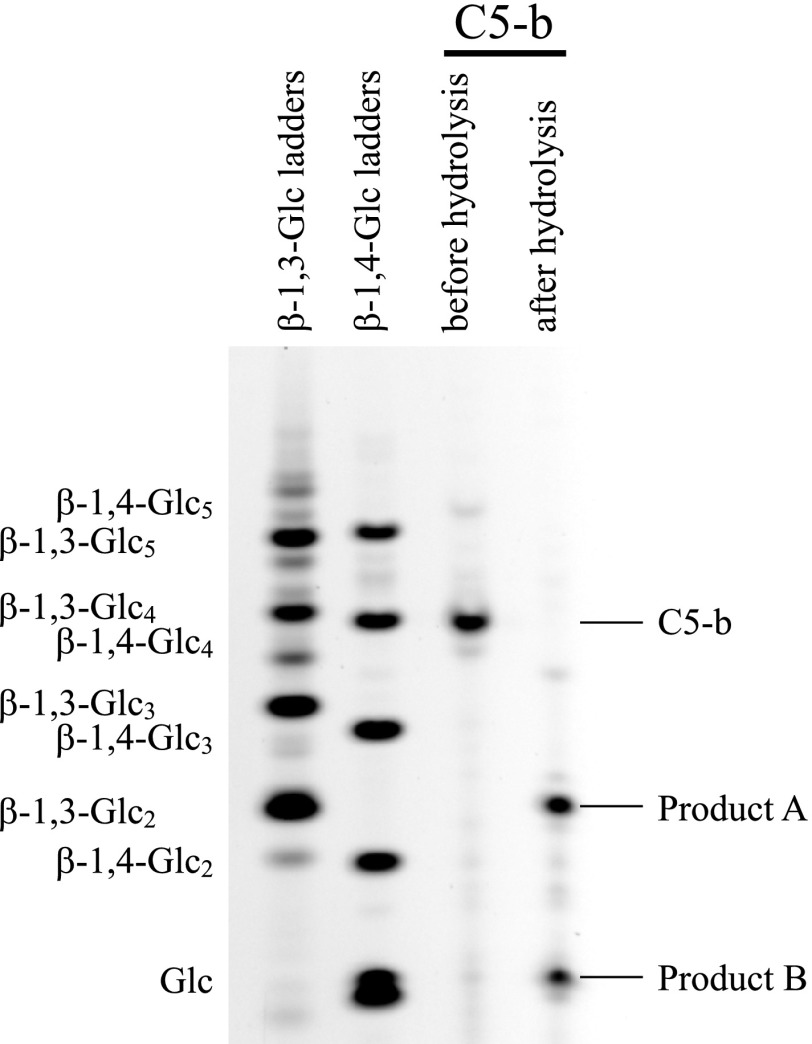
Analysis of products released from C5-b oligosaccharides by the *Trichoderma* enzyme on PACE.

Notes: Products released from C5-b by the *Trichoderma* enzyme were reductively aminated with 2-aminonaphthaline trisulfonic acid and separated by electrophoresis on acrylamide gels. A series of commercial laminarioligosaccharide (β-1,3-Glc_2–5_ and Glc) and that of cellooligosaccharide (β-1,4-Glc_2–5_ and Glc) were used in β-1,3-Glc ladder and β-1,4-Glc ladder markers, respectively. Product A corresponding to laminaribiose and B to Glc are indicated.

As shown in Fig. 3, *Trichoderma* enzyme properly hydrolyzed C5-b into smaller saccharides; however, the oligosaccharide could not be identified on HPLC. Therefore, the products released from C5-b were also subjected to PACE. As shown in Fig. 5, the smaller saccharides in the products were identified as laminaribiose and Glc. The result suggests that the enzyme acted on both β-1,4-linkages in G3G4G3G4G.

## Discussion

Poaceae β-1,3:1,4-glucan is mainly consisted of cellotriosyl and cellotetraosyl units linked through single β-1,3-glucosidic linkages, but it has also been shown to possess cellobiosyl units as the minor structure.^4^ ^)^ Through the structural analysis of unexpected oligosaccharides released by endo-β-1,3:1,4-glucanase, the cellobiosyl units appeared to locate at the nonreducing side of cellotriosyl units in barley, lichen, and horsetail.^5^ ^)^ Based on the proportion of the released oligosaccharides, the frequency of cellobiosyl unit was estimated less than 2% in barley β-1,3:1,4-glucan.^5^ ^)^ In this study, based on sugar content, C5 fraction obtained by digestion with *A. niger* endo-β-1,4-glucanase belonging to GH12 was less than 1.5% of total sugar released from barley β-1,3:1,4-glucan, confirming that the cellobiosyl units exist as the minor structure in barley β-1,3:1,4-glucan.

Together with cellobiosyl unit and long stretch of β-1,4-glucosidic linkage, continuous β-1,3-glucosidic linkages have also been presumed in maize β-1,3:1,4-glucan.^4^ ^)^ However, we could not detect any hydrolysis of barley β-1,3:1,4-glucan by barley endo-β-1,3-glucanases belonging to GH17, rGI, and rGII. The fact that laminaritriose is the smallest substrate for GII^22,37^ ^)^ suggests that barley β-glucan does not have three continuous β-1,3-glucosyl residues. Hence, the hydrolysis of β-1,3:1,4-glucan by endogenous GI and GII does not likely occur in barley. On the other hand, we cannot exclude the possibility that barley β-1,3:1,4-glucan has two continuous β-1,3-glucosyl residues that can be hydrolyzed by distinct endo-β-1,3-glucanases secreted by fungi and bacteria.

In the analysis of 3-D structure of PcLam16A,^20,21^ ^)^ two Trp residues have been shown to be involved in the specific recognition of β-1,3-glucosidic linkage between the subsites −1 and −2. In the enzyme, the substrate-binding cleft has a narrow and straight canyon structure along which a linear oligosaccharide such as G4G3G can lay. The *Trichoderma* enzyme hydrolyzed C5-b oligosaccharide, G3G4G3G4G, into laminaribiose and Glc as the final products. This result suggests that the *Trichoderma* enzyme either first hydrolyzed G3G4G3G4G into G3G4G3G and Glc, and then into two laminaribioses and Glc, or the enzyme first hydrolyzed G3G4G3G4G into G3G4G and laminaribiose, and then into two laminaribioses and Glc. Because the *Trichoderma* enzyme did not act on C4 oligosaccharide (G3G4G4G), the former case is more probable. These facts also suggest that the smallest substrate for the *Trichoderma* enzyme is G3G4G3G.

As well as endo-β-1,3(4)-glucanase, GH16 family comprises many carbohydrate active enzymes such as endo-β-1,3-glucanase, endo-β-1,3:1,4-glucanase, β-agarase (EC 3.2.1.81),^38^ ^)^ endo-β-1,3-galactanase (EC 3.2.1.181),^39^ ^)^ xyloglucan endo-transglycosylase/hydrolase,^40,41^ ^)^ and porphyranase (no EC entry).^42^ ^)^ On the basis of phylogenetic relationships, it has been proposed that an ancestral enzyme had endo-β-1,3(4)-glucanase (laminarinase) activity.^43,44^ ^)^ PcLam16A utilizes partially different subsites for β-1,3:1,4-glucan and β-1,3:1,6-glucan. Although the precise substrate specificity of the ancestral enzyme cannot been known, it is conceivable that the *Trichoderma* enzyme has adapted to cellobiosyl units as well as cellotriosyl and cellotetraosyl units in β-1,3:1,4-glucan keeping high activity toward β-1,3:1,6-glucan. The further study on 3-D structure of the *Trichoderma* enzyme would give an insight into the adaptation mechanism.

## Author contribution

 Takao Kuge, Kazufumi Tsubaki, Paul Dupree, Yoichi Tsumuraya, and Toshihisa Kotake conceived and designed the experiments. Takao Kuge, Hiroki Nagoya, Theodora Tryfona, Tsunemi Kurokawa, Yoshihisa Yoshimi, and Naoshi Dohmae performed the experiments. Hiroki Nagoya, Yoshihisa Yoshimi, Naoshi Dohmae, Theodora Tryfona, and Toshihisa Kotake analyzed data. Takao Kuge and Kazufumi Tsubaki contributed reagents/materials/analysis tools. Theodora Tryfona, Paul Dupree, Yoichi Tsumuraya, and Toshihisa Kotake wrote the paper.

## Supplemental material

The supplemental material for this study is available at http://dx.doi.org/10.1080/09168451.2015.1046365.

## Disclosure statement

No potential conflict of interest was reported by the authors.

## Funding

This work was supported in part by a grant-in-aid for Scientific Research to T. Kotake [Grant-in-Aid for Scientific Research no. 25514001] from Japan Society of the Promotion of Science; Y. Tsumuraya and T. Kotake [Grant-in-Aid for Scientific Research no. 24114006] from the Ministry of Education, Culture, Sports, Science, and Technology of Japan. Supports were also provided by BBSRC Sustainable Bioenergy Centre: Cell wall sugars program to P. Dupree [grant number BB/G016240/1].

## Supplementary Material

Supplemental Materials
