# Supplementary material for: Action of an endo-β-1,3(4)-glucanase on cellobiosyl unit structure in barley β-1,3:1,4-glucan
Source: Biosci Biotechnol Biochem. 2015 Jun 1;79(11):1810–7. doi: 10.1080/09168451.2015.1046365 (PMC4673573; doi:10.1080/09168451.2015.1046365)
Supplement: Supplemental Materials [file tbbb_a_1046365_sm2988.zip › 1046365_Supp/GucanOligoSupportInfo150224.docx]

**Supplemental information**

Preparation of rGI and rGII

*cDNA cloning of barley endo-β-1,3-glucanases GI and GII.* Total RNA was extracted from seedlings of barley (cv. Shikokuhadaka 97) with an Isogen kit (Nippon Gene, Tokyo, Japan) according to the manufacturer’s instruction. Single-strand cDNA was synthesized from approximately 1 µg of the total RNA using a reverse-transcriptase, ReverTra Ace-*α*- (Toyobo, Osaka, Japan), and oligo(dT)_12-18_ (Invitrogen, Carlsbad, CA, USA) as described.^1)^ With sets of specific primers designed based on the amino acid sequences of GI and GII,^2)^ GI-F (5'-GCGAATTCCATHGGNGTNTGCTACGGCGTG-3') and GI-R (5'-GGTTCTAGACTACTGGAACCGGATGGGG-3'), and GII-F (5'-GCGAATTCCATCGGCGTGTGCTACGGC-3') and GII-R (5'-GGTTCTAGACTAGAACTGGATGTTGTATGC-3'), the cDNA fragments encoding mature forms of GI and GII were amplified with KOD-Plus (Toyobo), respectively. The cDNA fragments were digested with EcoRI and XbaI, and then subcloned between the EcoRI site and XbaI site that are preceded by yeast α-factor of pPICZαC (Invitrogen).

*Heterologous expression of enzymes in* P. pastoris*.* The methylotrophic yeast *P. pastoris* strain KM71 was transformed with the linearized plasmid constructs for GI and GII with a multicopy *Pichia* expression kit (Invitrogen). The transformants resistant to zeocin were screened according to the manufacturer's instruction. The zeocin resistent colony was cultured in 800 mL of YPG medium containing 1% (w/v) yeast extract, 2% (w/v) peptone and 1% (w/v) glycerol at 30°C with shaking at 100 rpm for 2 days. The cells were harvested by centrifugation at 3,000 rpm for 5 min, washed with ice-cold distilled water, and then suspended in 200 mL of YPM medium containing 1% (w/v) yeast extract, 2% (w/v) peptone, and 1% (v/v) methanol. The yeast cells were cultured for another 4 days at 30°C, during which time 2 mL of methanol was added each day, to induce the recombinant enzymes. The recombinant GI (rGI) and rGII were purified by the same purification procedures. The culture medium of the *Pichia* cells including recombinant enzyme was centrifuged at 8,000 rpm for 15 min, and the supernatant was collected as a crude enzyme fraction. The crude enzyme fraction was dialyzed against 10 mM sodium acetate buffer (pH 5.0), and then first applied to a Toyopearl HW75 (Tosoh, Tokyo, Japan) column to remove pigments. The sample was adsorbed onto a CM-Sepharose Fast Flow (GE Healthcare, Buckinghamshire, UK) column that had been equilibrated with the buffer. The recombinant enzyme was eluted with a linear gradient of 0-500 mM NaCl in the buffer. The purity of the recombinant enzymes was determined on SDS-PAGE (Supplemental Fig. 1).^3)^ The enzyme in the gel was stained with Coomassie Brilliant Blue R-250.

References

1. Hrmova M, Fincher GB. Purification and properties of three (1→3)-β-D-glucanase isoenzymes from young leaves of barley (*Hordeum vulgare*). Biochem. J. 1993;289:453-461.
2. Kotake T, Kaneko S, Kubomoto A, Haque MA, Kobayashi H, Tsumuraya Y. [Molecular cloning and expression in *Escherichia coli* of a *Trichoderma viride* endo-β-(1→6)- galactanase gene.](http://www.ncbi.nlm.nih.gov/pubmed/14565843) Biochem. J. 2004;377:749-755.
3. Laemmli UK. Cleavage of structural proteins during the assembly of the head of bacteriophage T4. Nature 1970;227:680-685.
